# Metronomic cyclophosphamide for bone marrow carcinomatosis in metastatic castration-resistant prostate cancer

**DOI:** 10.1007/s00432-023-05525-0

**Published:** 2024-02-08

**Authors:** Tobias Peres, Stefanie Aeppli, Stefanie Fischer, Katrin Gysel, Christian Rothermundt

**Affiliations:** 1 Department of Medical Oncology and Hematology, Cantonal Hospital St. Gallen, Rorschacher Strasse 95, 9007 St. Gallen, Switzerland; 2 Competence Center of SAKK, Bern, Switzerland

**Keywords:** Metronomic cyclophosphamide, Bone marrow carcinomatosis, Castration resistance, Prostate cancer, Thrombocytopenia

## Abstract

**Purpose:**

In some patients with prostate cancer, bone marrow carcinomatosis develops later in the course of the disease, which has a poor prognosis. These are often heavily pretreated patients in the castration-resistant situation for whom there are no other therapeutic options, because either all available systemic therapies have already been used or the use of one is not possible due to the cytopenias associated with bone marrow carcinomatosis. In our literature search, there are no data on this treatment in the setting available, especially no clinical trial or even randomized data. This case series is to determine the clinical efficacy of metronomic cyclophosphamide in patients with metastatic castration-resistant prostate cancer and bone marrow carcinomatosis, particularly with regard to stabilization of the blood count (thrombocytopenias) and thus the possibility of further (more toxic) lines of therapy.

**Methods:**

Retrospective unicenter analysis was performed on eleven patients between 54 and 84 years of age on metronomic cyclophosphamide for bone marrow carcinomatosis in metastatic castration-resistant prostate cancer treated at a Swiss cancer center between 2014 and 2023.

**Results:**

Eleven patients received metronomic cyclophosphamide for varying periods of time; the majority had severe cytopenias (especially thrombocytopenias). Partially hematologic stabilization was achieved with administration of further systemic therapies.

**Conclusion:**

Our case series demonstrates that the use of metronomic cyclophosphamide allows hematologic stabilization for months, benefiting patients who had already received all available therapies for metastatic castration-resistant prostate cancer. Alternatively, it may act as bridging therapy to allow consecutive administration of more toxic therapies with proven survival benefit.

## Introduction: metronomic chemotherapy in metastatic castration-resistant prostate cancer (mCRPC)

Docetaxel was the first chemotherapy to show a survival benefit for patients with mCRPC (Tannock 2004). Mitoxantrone in combination with prednisone had previously been demonstrated to improve pain control and quality of life, respectively, without any survival benefit compared to prednisone (Tannock 1996).

The use of metronomic chemotherapy, i.e., low doses given continuously every day, was also investigated in the 1990s, at a time when there were no broad therapeutic alternatives for patients with mCRPC. Rationale is to effectively expose the predominantly low-proliferative neoplasia of mCRPC to low-dose continuous therapy. Most commonly, metronomic cyclophosphamide is used and has been studied in various combinations, including corticosteroids, and as monotherapy. Metronomic cyclophosphamide has (as an alkylating agent) direct cytotoxic, anti-angiogenic (e.g. reduction of VEGF levels in plasma) (Kerbel 2004; Duque 1999; Fontana 2009) and immune-modulating effects (Rozados 2010; Ghiringhelli 2006). Cyclophosphamide targets not only the tumor cells themselves but also the vascularization of the tumor environment and is thus expected to overcome potential drug resistance. In addition, the relatively low single dose entails good subjective tolerability (Gnoni 2015).

After the establishment of various chemotherapies and new hormonal agents, such as androgen receptor pathway inhibitors (ARPIs), the use of metronomic therapy regimens remains limited to patients who have already received all standard therapies, primarily with the aim of at least stabilizing the advanced disease by means of a well-tolerated and inexpensive medication.

For example, Caffo et al. retrospectively demonstrated a PSA response (defined as > 50% reduction vs. baseline) in 12 out of 74 pretreated patients with mCRPC (after docetaxel and at least 1 new agent (NA), i.e., abiraterone, enzalutamide, cabazitaxel, or radium 223; the majority had received 1–2 NA), corresponding to 16% of the population. Cyclophosphamide was given as monotherapy with 50 mg daily (Caffo 2019). Four patients showed a partial radiographic response. The median progression-free survival was 4 months, and the median overall survival was 8.1 months, respectively. It is also emphasized by the authors that 13 patients remained progression-free for at least 6 months, including 2 patients for more than 12 months. Based on this activity of metronomic cyclophosphamide the authors also draw a comparison to new hormonal agents, which have been studied in third or fourth line therapy in mCRPC (Caffo 2015).

The main toxicities were grade 1–2 according to common terminology criteria of adverse events (CTCAE): nausea, hyporexia, fatigue, and cytopenias (especially anemia). Treatment was thus relatively well tolerated.

Calvani et al. retrospectively analyzed 37 patients with mCRPC, including those heavily pretreated but also unfit for standard chemotherapy/NA (Calvani 2019). All patients received androgen deprivation therapy (ADT), and 62% were pretreated with docetaxel. Patients received 50 mg cyclophosphamide plus dexamethasone 1 mg (21 patients; 57%) or prednisone 10 mg daily. Primary endpoint was a decrease in PSA concentration by at least 50%. More than half of the patients had an ECOG 2–3, median age was 75 years. Seventeen patients (51%) showed a PSA response > 50%. Median progression-free survival in the overall population was 14, median overall survival was 28 months (for the subgroup with PSA response > 50% 14 and 35 months, respectively). Among the 13 patients who had not had prior docetaxel therapy, nine (69%) had a PSA response > 50%, median progression-free and overall survival being 19 and 35 months, respectively. However, few patients were included who had received a therapy sequence that would nowadays be considered a standard: two of seven (28.5%) patients who received metronomic cyclophosphamide after docetaxel and abiraterone and/or cabazitaxel showed a PSA response > 50%. Overall, metronomic cyclophosphamide was very well tolerated, no patient had to discontinue treatment due to toxicity, only one patient had a dose reduction (50 mg three times per week).

However, the strength of evidence for the use of metronomic cyclophosphamide in prostate cancer is limited by the lack of prospective phase 3 data. The cohorts studied in the published predominantly single-arm trials are mostly small and relatively heterogeneous (Parshad 2022).

## Introduction: disseminated carcinomatosis of bone marrow

Metastatic solid tumors are variably associated with prognostically unfavorable bone marrow involvement (Kucukzeybek 2014). Common solid tumors associated with bone marrow carcinomatosis include prostate carcinoma, gastric carcinoma, breast carcinoma, and lung carcinoma. (Mehdi 2011; Arya 2021; Tyagi 2018). In the literature, the term disseminated bone marrow carcinomatosis is used to illustrate the diffuse infiltration of the bone marrow by tumor cells. This is often associated with clinically relevant hematologic and hemostaseologic pathologies (including cytopenias and disseminated intravascular coagulation (DIC)) (Hayashi 1979; Shinden 2018; Duran 2006).

In prostate cancer, evidence of bone marrow infiltration by adenocarcinoma cells can be obtained cytologically as well as histologically (Shahait 2022). Usually metastasis to bone marrow occurs late in the course of a castration-resistant situation. This results in myelosuppression: typically, cytopenias are seen in the peripheral blood, although these are not specific for bone marrow carcinomatosis (Massenkeil 2017). Of note, for thrombocytopenia—and especially severe thrombocytopenia corresponding to values < 50/nl—a shortened overall survival is explicitly described in the literature. Reasons for the poor prognosis are potential fatal hemorrhages and the impossibility to administer antineoplastic chemotherapy (Kilickap 2007).

On the other hand, due to the impaired medullary-blood barrier, a leucoerythroblastic blood count with continuous left shift and detection of erythroblasts and granulocyte precursors (including myelocytes) is found in the peripheral blood (Shamdas 1993). In this context, it should be mentioned that in clinical practice, a leucoerythroblastic blood count is considered to be sufficiently diagnostic for the presence of bone marrow carcinomatosis when possible differential diagnoses, including sepsis, have been appropriately excluded. (Mahdi 2014; Sheng 2019). Literature reports include an incidence for leucoerythroblastic blood count in mCRPC of nearly 30% (Shamdas 1993). Logically, the incidence of bone marrow carcinomatosis can be expected to increase as new survival-prolonging therapies become available. Furthermore, it can be assumed that this differential diagnosis is considered more frequently by the treating physicians due to greater attention and knowledge. However, there are virtually no reliable data on the development of the incidence of bone marrow carcinomatosis in prostate cancer in the literature.

**Fig. 1:**
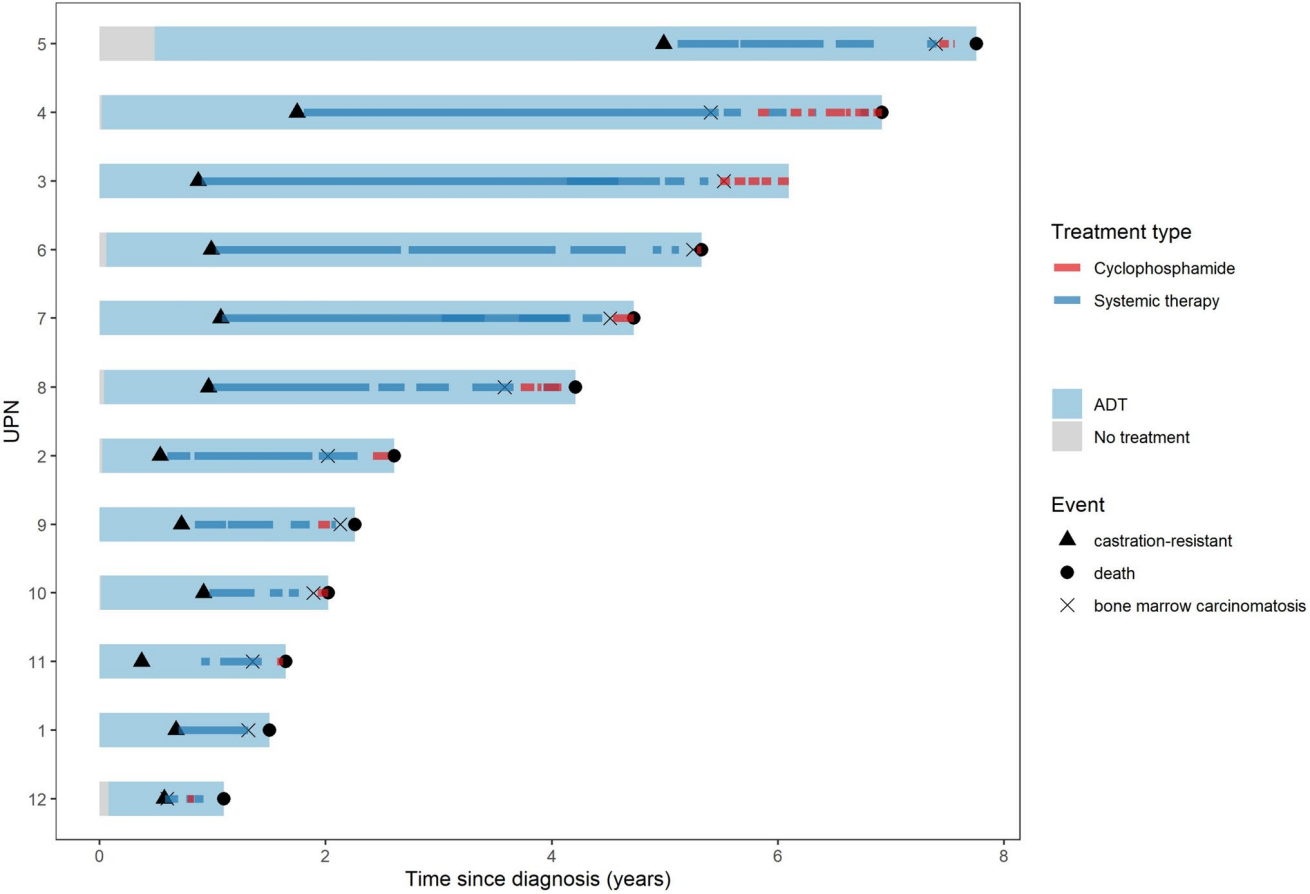
Timeline with applied systemic therapies including cyclophosphamide and onset of castration resistance, bone marrow carcinomatosis and death of 12 patients

The swimmer plot (Fig. 1) shows 12 patients with advanced prostate cancer and bone marrow carcinomatosis who were treated at our Cancer Center from 2014 to 2023. One patient did not receive metronomic cyclophosphamide (see Case Report UPN 1). The other patients were treated with metronomic cyclophosphamide for varying periods of time. As can be seen, all but one patient died. Furthermore, in addition to ADT, periods of systemic therapy applied in the presence of castration resistance, including metronomic cyclophosphamide, are shown.

## Case report—no treatment for bone marrow carcinomatosis (UPN 1)

**Fig. 2:**
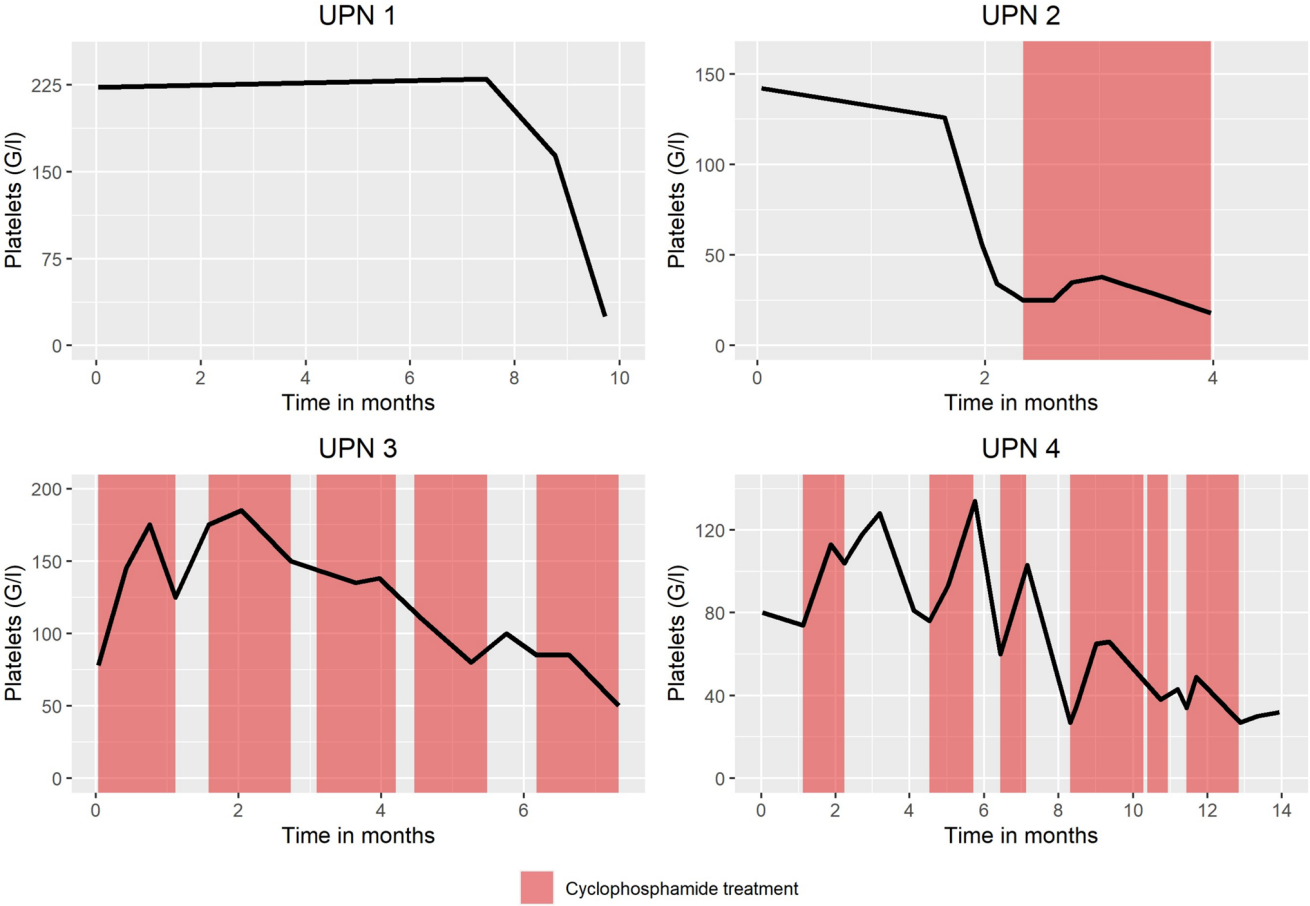
Platelet courses UPN 1 (no cyclophosphamide), UPN 2 (short-term cyclophosphamide), UPN 3 (long-term cyclophosphamide), UPN 4 (intermittent cyclophosphamide)

The poor prognosis in the presence of bone marrow carcinomatosis or thrombocytopenia is illustrated by a patient who was treated in our Cancer Center. This 77-year-old patient was diagnosed with secondary metastatic hormone-sensitive prostate carcinoma in 03/2020. In accordance with the guidelines, ADT was initiated and intensification of therapy using ARPI available at that time was declined by the patient. Disease progression in terms of castrations resistance occurred as early as 11/2020. Subsequently, the ARPI enzalutamide was started. However, enzalutamide was discontinued again in 06/2021 due to clinical, laboratory and progression on imaging. At this time, severe anemia (Hb 72 g/l) was detected, and differential microscopic blood counts showed metamyelocytes consistent with bone marrow carcinomatosis for the first time. In addition, leukopenia (3.2 G/l) appeared at the end of 07/2021, and severe thrombocytopenia (25/nl) at the end of 08/2021 (see Fig. 2). No further antineoplastic therapy was given. Death occurred 1 week after the onset of thrombocytopenia. Accordingly, this patient died only about 8 weeks after the first detection of a leucoerythroblastic blood picture and illustrates impressively the prognostically poor spontaneous course in the presence of bone marrow carcinomatosis.

### Metronomic cyclophosphamide in bone marrow carcinomatosis

According to our literature search (PUBMED, MEDLINE), there is no publication to date on the use of metronomic chemotherapy in mCRPC with concurrent bone marrow carcinomatosis. Here, we will retrospectively present the course of three patients (UPN 2, 3, 4) from our Cancer Center who received metronomic cyclophosphamide in combination with steroids for an already extensively pretreated mCRPC.

## Case report—short-term treatment with metronomic cyclophosphamide for bone marrow carcinomatosis (UPN 2)

The potential of metronomic cyclophosphamide to stabilize the blood count in patients with disseminated bone marrow carcinomatosis is evident from another case at our Cancer Center when considering platelet progression. In 07/2020, a primary metastatic prostate carcinoma was initially diagnosed in a then 74-year-old patient. After initiation of ADT and intensification with docetaxel for hormone-sensitive disease, disease progression and corresponding castration resistance occurred in 01/2021. Subsequently, enzalutamide, olaparib (in the presence of a somatic BRCA2 mutation), ^177^lutetium-PSMA, and cabazitaxel were administered sequentially. In 7/2022, peripheral microscopic blood counts showed myelocytes and metamyelocytes, so that the presence of bone marrow carcinomatosis had to be assumed. A bone marrow biopsy performed later, in 12/2022, histologically confirmed the infiltration of the bone marrow with prostate carcinoma cells. At that time, severe pancytopenia was already present. In addition, there was clear evidence of DIC with prolonged prothrombin time/partial thromboplastin time and increased D-dimer concentration. Metronomic cyclophosphamide (50/100 mg alternating)/dexamethasone (2 mg continuously) was administered from 12/2022, resulting in a slight improvement and at least stabilization of the blood count over 4–5 weeks, illustrated by the platelet trend (see Fig. 2). However, from 01/2023 onwards there was again a drop in the platelet values with death of the patient shortly thereafter.

## Case report—long-term treatment with metronomic cyclophosphamide for bone marrow carcinomatosis (UPN 3)

However, longer-term hematologic stabilization over months is also possible with metronomic cyclophosphamide, as shown by platelet counts over time of the following case. In 2017, a then 62-year-old patient at our Cancer Center was newly diagnosed with primary metastatic prostate carcinoma. Imaging showed disseminated osseous metastases in addition to non-regional lymph node metastases. Initially, the patient received ADT and intensification with docetaxel. At progression, castration resistance was formally present as of 12/2017. In this setting, the patient received successive enzalutamide, ^177^lutetium-PSMA, a re-exposure with enzalutamide, later cabazitaxel as well as radium 223 most recently in 06/2022. Myelocytes were detectable for the first time in the microscopic differential blood picture, so that a bone marrow carcinomatosis was postulated. Pancytopenia was present (platelets 78 G/l, Hb 101 g/l, leukopenia 2.9 G/l). In addition, there was clear evidence of activated coagulation with increased D-dimer concentration and prolonged prothrombin time in this case as well. Bone marrow aspiration to confirm the diagnosis of bone marrow carcinomatosis was not performed. In the absence of other therapeutic options, metronomic chemotherapy with cyclophosphamide at a dose of 50 and 100 mg alternated, with concomitantly continuous dexamethasone 1 mg, was administered starting in 07/2022. Platelet levels stabilized after 2 weeks of treatment (see Fig. 2). Over the course of the following months, cyclophosphamide treatment was interrupted and resumed several times because of suspected hematotoxicity with decreasing leucocyte counts after prolonged cyclophosphamide exposure. In 03/2023, there was also an exacerbation of anemia and leukopenia. Regarding the coagulation situation, there was an improvement in D-dimer and prothrombin time on 08/11/2022, followed by a significant increase in D-dimer from 02/02/2023. Cyclophosphamide was stopped definitely in March 2023. As of end of May 2023, the patient is still alive and undergoing best supportive care requiring regular transfusions of erythrocytes and platelets about every second week.

## Case report—intermittent treatment with metronomic cyclophosphamide for bone marrow carcinomatosis (UPN 4)

The following case study demonstrates that metronomic cyclophosphamide can also be used to enable a more hematotoxic antineoplastic therapy through repeated—at best, short term—applications with appropriate breaks. In 2014, a then 78-year-old patient at our Cancer Center was newly diagnosed with primary osseous and lymphogenic metastatic prostate carcinoma, which was initially treated with ADT only. After progression to castration-resistant disease in 2016, the patient received enzalutamide/metformin as part of a clinical trial and later radium 223. Myelocytes were already detectable in the microscopic blood count as of 02/2020 as an indicator of bone marrow carcinomatosis. From 07/2020 onwards metronomic cyclophosphamide 50 mg (plus 1 mg dexamethasone) was started, which was subsequently used intermittently for more than 1 year. Bicytopenia was already present in 07/2020 (platelets 77 G/l, Hb 110 g/l), and there was also evidence of DIC with a markedly increased D-dimer level. During the cyclophosphamide breaks, radium 223 (08/10/2020), docetaxel (12/2020), and ^177^lutetium-PSMA (01/2021) could be administered. In 06/07/2021, a parallel re-exposure to enzalutamide was performed under ongoing cyclophosphamide treatment. The presented graph shows that after start of metronomic cyclophosphamide, there was an initial increase in platelets followed by stabilization of values over several weeks (see Fig. 2). The platelet decrease in 09/10/2020 occurred under pause of cyclophosphamide but continued application of radium223, so that primarily hematotoxicity caused by the latter treatment was assumed. The respective platelet increases due to repeated re-exposure to cyclophosphamide at the beginning of 11/2020 (after radium 223), at the end of 12/2020 (after docetaxel) and at the end of 02/2021 (after ^177^lutetium-PSMA) are also graphically tractable and reflect the potential of cyclophosphamide to stabilize the blood count and thus enable a re-administration of a potentially more hematotoxic (chemo)therapy.

In 6 out of 11 patients, an improvement in the blood count can be seen under metronomic cyclophosphamide (UPN4, UPN3, UPN6, UPN7, UPN8, UPN2). In 3 patients, cyclophosphamide did not cause any obvious improvement in the blood count (UPN5, UPN10, UPN11). In two patients, at least a stabilization of the blood count under cyclophosphamide was observed (UPN9, UPN12). (Table 1).

**Table 1:** Blood value changes between baseline (diagnosis of bone marrow carcinomatosis) and best response under cyclophosphamide therapy (shown per UPN for Hb hemoglobin, PLT platelets, Lc leukocytes; medians and interquartile ranges IQR in the bottom row)

UPN	Baseline			Best response under Cyclophosphamide			Cyclophosphamide therapy duration
	Hb [g/l]	PLT [G/l]	Lc [G/l]	Hb [g/l]	PLT [G/l]	Lc [G/l]	[months]
5	76	71	2.3	93	48	2.6	1.6
4	110	77	4	114	134	7.7	13.1
3	101	78	2.9	111	192	4.1	7.3
6	82	24	13.3	88	67	13.4	0.5
7	65	74	5.1	74	104	3.9	2.2
8	68	66	3.8	98	119	4.3	5.8
2	93	78	7.6	93	177	9.4	1.6
9	120	212	3.8	111	289	5.1	1.5
10	96	55	7.5	104	44	6.1	1.1
11	98	54	4.7	93	43	4.3	0.7
12	77	134	7.9	80	139	69	0.7
Median (IQR)	87.5 (75, 98.8)	75.5 (63.3, 92)	4.9 (3.8, 7.5)	93 (90.5, 107.5)	119 (57.5, 158)	5.1 (4.2, 8.6)	1.6 (0.9, 4)

Fourth column shows the average duration of cyclophosphamide treatment

## Discussion

This is a case series of patients with mCRPC and bone marrow carcinomatosis. Our first case illustrates the rapid fatal spontaneous course of a patient with advanced mCRPC after bone marrow carcinomatosis was diagnosed. Without treatment, death occurred after only a few weeks. In the second case report, the patient had already received all available therapies in the castration-resistant setting. After failure of cabazitaxel, increasing pancytopenia occurred, and bone marrow carcinomatosis was confirmed histologically. In this disease setting, only cyclophosphamide (in combination with dexamethasone) remained as a therapeutic option. The stabilization of the platelet values (albeit at a low level), which can be traced graphically, may even indicate a prolongation of survival by cyclophosphamide. In the third case report, hematologic stabilization is evident for at least 5 months in a heavily pretreated patient. Of particular note, the patient had a very good subjective quality of life and no relevant treatment related side effects or tumor symptoms, underscoring the practicality of the cyclophosphamide medication in a heavily pretreated patient with end-stage disease. The fact that cyclophosphamide can be used judiciously in the castration-resistant setting even before current therapy options is demonstrated by the patient in the fourth case report. Already after the use of enzalutamide/metformin, a relevant bicytopenia with leucoerythroblastic blood count was detectable, whereby a conventional and potentially hematotoxic chemotherapy was not feasible. Cyclophosphamide improved the blood count, so that radium 223, docetaxel and ^177^lutetium-PSMA could finally all be administered sequentially. Cyclophosphamide was paused for the corresponding treatment phases. Thus, in this case study, cyclophosphamide acted as a bridging therapy to allow the use of established hematotoxic (chemo)therapies with proven survival benefits. We conclude that the use of metronomic cyclophosphamide in combination with dexamethasone can result in hematologic stabilization over a (short) period of time.

The main limitation of this case series is the small sample size. However, we are not aware of any other publication on this topic, nor of any clinical study or even randomized data.

There are reports on bone marrow carcinomatosis in other tumor types, e.g. breast cancer and gastrointestinal cancers. According to the guidelines, chemotherapy is generally recommended for HR + HER2- breast carcinomas with rapid disease and visceral involvement, as a faster response to chemotherapy is expected than to endocrine therapy. A diagnosis of clinically relevant bone marrow carcinomatosis in breast carcinoma is ultimately rare. Again, prognosis is poor, and such patients are virtually unrepresented in studies. However, cases have been described in the literature in which patients received continuous endocrine therapy and CDK4/6 inhibitors and achieved a sufficient, long-lasting response, e.g. a 46-year-old premenopausal patient who underwent a bone marrow aspiration for pancytopenia, which revealed infiltration by a poorly differentiated HR + /HER2- lobular breast carcinoma. As the patient refused chemotherapy, the aromatase inhibitor letrozole was administered in combination with the CDK4/6 inhibitor palbociclib (in addition to leuprorelin in premenopause). Although this resulted in intermittent neutropenia (class effect of CDK4/6 inhibitors), the blood count otherwise normalized after a few months, with the known metastases also showing a response on PET/CT (Garufi 2021). Chemotherapy in bone marrow carcinomatosis-related cytopenias would certainly entail a higher potential for complications with poorer subjective tolerability. Among gastrointestinal tumors, gastric carcinoma is most likely to be associated with bone marrow carcinomatosis. However, there are also case reports of pancreatic carcinoma with bone marrow carcinomatosis, so that even this primary tumor must be considered for differential diagnosis if bone marrow carcinomatosis is detected. For example, the literature describes the case of a 57-year-old patient who had DIC in conjunction with various bone metastases. A bone marrow biopsy showed infiltration by an adenocarcinoma, with CT evidence of a mass in the tail of the pancreas consistent with infiltration by an adenocarcinoma of the pancreas (Namikawa 2016).

In the treatment of prostate cancer, the trend has been to use established drugs earlier in the disease course and to combine different agents rather than using them sequentially (Freedland 2021; James 2017; Smith 2022), and this strategy has led to clinically relevant improvements in overall survival. Nonetheless, mCRPC remains a deadly disease and some patients will live to experience the development of bone marrow carcinomatosis. Patients with a good quality of life may present with this severe clinical problem at a time when all standard treatment options are exhausted or cannot be applied.

Hence, we believe that metronomic cyclophosphamide remains a well-tolerated option for the treatment of prostate cancer with bone marrow carcinomatosis, even in times of many innovative new therapies.

## Data Availability

The datasets generated during and/or analysed during the current study are available from the corresponding author on reasonable request.
